# Blood-borne and sexually transmitted infections: a cross-sectional study in a Swiss prison

**DOI:** 10.1186/s12879-018-3445-6

**Published:** 2018-10-29

**Authors:** Komal Chacowry Pala, Stéphanie Baggio, Nguyen Toan Tran, François Girardin, Hans Wolff, Laurent Gétaz

**Affiliations:** 1 Division of Prison Health, Geneva University Hospitals, University of Geneva, Chemin de Champ-Dollon 22, 1241 Puplinge, Geneva, Switzerland; 2 Medical Direction and Division of Clinical Pharmacology, Toxicology Geneva University Hospitals, University of Geneva, Geneva, Switzerland; 3 Division of Tropical and Humanitarian Medicine, Geneva University Hospitals, University of Geneva, Geneva, Switzerland; 4 Australian Centre for Public and Population Health Research, Faculty of Health, University of Technology, Sydney, Australia

**Keywords:** HIV, Syphilis, Herpesvirus 2, human, Hepatitis C, Prison, Epidemiology

## Abstract

**Background:**

Incarcerated people carry a high burden of infection, including blood-borne diseases (BBDs). It is also known that one million people contract a sexually transmitted infection (STI) every day worldwide, which represents a global public health challenge. However, data regarding the prevalence of STIs and the risk factors among incarcerated populations are lacking. The objective of this study was to determine the prevalence and associated factors of BBDs and STIs among detainees in the largest pre-trial prison in Switzerland.

**Methods:**

In a cross-sectional study conducted at the Champ-Dollon pre-trial prison, 273 male detainees answered a standardized questionnaire and were screened for syphilis, herpes simplex virus 2 (HSV-2), HIV, and hepatitis C (HCV). Prevalence rates and associations of BBDs and STIs with risk factors were computed.

**Results:**

Most participants (90.9%) were migrants from outside Western Europe, and 5.9% were injecting drug users. HCV was diagnosed among 6.2% of participants (antibody prevalence). The prevalence of HCV was higher among injecting drug users (81.2%) than non-injectors (1.6%). The prevalence of HIV, syphilis, and HSV-2 was 0.4%, 1.1%, and 22.4%, respectively. HCV was associated with a history of injecting drug use and HSV-2 with a lower education level and being older than 26 years.

**Conclusions:**

This study showed the infection prevalence of 2–9 times higher among detainees than in the Swiss community. It also illustrated that these infections are associated with sociodemographic and risk factors. Therefore, the prison environment offers an opportunity to strengthen infectious disease control programs targeting specific subgroups of at-risk people. Such programs would benefit both the prison population and broader society.

## Background

### Blood-borne and sexually transmitted infections in prison

Incarcerated people are members of a vulnerable group and carry a high burden of medical conditions, including infectious diseases [1, 2]. Furthermore, precarious living conditions in jails, such as a sedentary lifestyle, a poor diet, inadequate hygiene habits, and drug use, contribute to the transmission of infectious diseases [3]. In prisons, high prevalence rates of blood-borne diseases (BBDs) and sexually transmitted infections (STIs) may be related to the accumulation of negative health risks. Due to the long asymptomatic periods, the prevalence rate of STIs could also be related to the epidemiology reported in detainees’ countries of origin, where they spent part of their lives and where they acquired most of their infections prior to the migration process [4]. Approximately one million people contract an STI every day worldwide. Despite progress in diagnosis, treatment, and prevention, STIs continue to represent a global public health challenge with substantial socio-economic burden [5].

Syphilis and herpes simplex virus 2 (HSV-2) are known to be major global causes of acute illness and long-term disability and cause significant complications [5]. Both can also increase the risk of acquiring and transmitting other infections [6], particularly HSV-2, which is associated with an increased risk of HIV acquisition by two- to threefold [7, 8]. Since 2010, many countries, particularly in Western Europe, saw a sharp upsurge in the number of reported syphilis infections [9], which is a public health concern, as 15% of people infected with latent syphilis will present with severe complications if left untreated [9]. Data regarding the prevalence of STIs and risk factors among incarcerated populations are missing [10]. HIV and HCV affect millions of people around the globe and cause profound morbidity and mortality [11]. Of the estimated 10.2 million people incarcerated worldwide, it is reported that 3.8% are infected with HIV (389,000 living with HIV) and 15.1% with HCV (1,546,500) [12]. This prevalence is much higher than in the general population, primarily because of high-risk behaviors, in particular, sharing needles to inject drugs [12, 13].

Correctional facilities are known to provide a valuable and unique opportunity to offer accessible and acceptable health interventions [12]. Therefore, identifying and treating incarcerated people carrying BBDs and STIs may not only limit the disease burden and increased costs by means of early treatment, but also contribute to decrease the overall transmission of STIs, HCV, and HIV in the general population upon their release from prison [14].

### The case of Switzerland

Our study took place in Switzerland, which has a prison population of 6,869 people, among whom approximately 71% are foreigners [15]. These foreign detainees come mostly from poor and vulnerable communities [16] and may suffer from a higher burden of disease compared to Swiss prisoners. Undocumented detainees do not have any health insurance coverage and therefore have limited access to health care when living in Switzerland. This lack of health care access includes the access to vaccination, diagnosis and treatment of HIV, HCV, syphilis, and HSV-2.

In Switzerland, Champ-Dollon prison is the largest and most overcrowded (177%) pre-trial prison [17]. It accommodated, on average, 540 prisoners between 2009 and 2011. This prison is also characterized by a high turnover rate of detainees (73%) [17]; approximately half of the prisoners are released within one month of initial incarceration. The medical unit of this prison is attached to Geneva University Hospitals and is completely independent of the prison’s administration. It offers a low-threshold primary care approach to health care. All detainees admitted to the facility undergo a health assessment by primary health care nurses. This evaluation acts as triage to identify any health problems that require medical attention, such as allergies; injuries; breathing problems; mental health problems, including suicidal ideas; addiction; regular medical treatment; suspicion of tuberculosis; or allegations of violence during arrest. When necessary, nurses immediately refer detainees to a primary care physician. A proposal for screening for infectious diseases (hepatitis, HIV, syphilis) is discussed at this time, but these problems are often not considered a priority by inmates at the time of entry into prison. In case of initial refusal, as these screenings are not systematically proposed again later during incarceration, a large proportion of prisoners does not benefit from these diagnoses before being released. In Geneva’s prisons, when detainees are diagnosed as positive, they are treated in accordance with good medical practices, respecting the principle of equivalence of care and corresponding to the care provided to people who are free [18]. Regarding the prevention of infectious disease transmission in this prison, specific harm-reduction measures, such as needle and syringe exchange programs, condom distribution, and opioid substitution treatment are provided [19, 20].

### Study’s objectives

This study aims to determine the prevalence of syphilis, HSV-2, HIV, and HCV serological markers and their associated factors among detainees in the Champ-Dollon prison in Geneva, the largest pre-trial detention center in Switzerland.

## Methods

A cross-sectional study was conducted at the Champ-Dollon prison during two varicella outbreaks, which occurred in the male detention units at two distinct times (2009 and 2011).

### Study population and setting

Capitalizing on the blood samples taken to test for varicella immunity in all varicella-exposed detainees, we offered these same consenting participants the opportunity to test for HIV, HCV, and syphilis (HSV-2 was only offered to the 2011 cohort) and to take a structured socio-demographic survey. We offered consenting participants of entire affected prison units the opportunity to participate. Women, representing 5% of the prison population in Champ-Dollon, are detained in a separate unit. Since the varicella outbreaks did not affect this unit, all participants were men. We made sure that detainees were not included twice in the study (i.e., in case of re-arrest). There were no exclusion criteria. Of the 281 detainees, 273 agreed to participate in the study (*n* = 116 in 2009, and *n* = 157 in 2011; participation rate = 97.2%).

### Ethics statement

The Ethics Research Committee of the Geneva University Hospitals approved the study (EC: 09–137).

### Data collection

Each participant underwent serological testing for HIV, HCV, and syphilis. One participant refused the HIV serology. Serum was insufficient to process HSV-2 in one participant and syphilis and HIV in two participants. HSV-2 was tested for only among participants recruited in 2011 (n = 157). Serum samples were tested by commercial immunoenzymatic assays for HIV (HIV, Ag/Ab Combo, Architect, Abbott) and HCV (anti-HCV, Architect, Abbott). Anti-HIV and anti-HCV reactivity were always confirmed via INNO-LIA™ HIV I/II Score and INNO-LIA™ HCV score line immune assays (Innogenetics, Belgium), respectively. HSV-2 serostatus was determined using an HSV-2 IgG enzyme-linked immunosorbent assay (ELISA) (Immunowell, HSV type 2, IgG test, GenBio). A positive HSV-2 serology indicates present or past infection. Syphilis was screened using a two-step Chemiluminescent Microparticle ImmunoAssay (CMIA) (Syphilis TP, Architect, Abbot). Positive cases were confirmed via Treponema Pallidum Particle Agglutination (TPPA) (Serodia®) and the serum Rapid Plasma Reagin (RPR) test (Human). Those CMIA+/RPR+/TPHA+ and without a history of syphilis treatment were assumed to have syphilis. Participants answered a standardized questionnaire administered by a physician. Questions (provided in six different languages: English, French, German, Italian, Spanish, and Russian) explored socio-demographic characteristics (age, sex, country of birth, self-reported socioeconomic status, and education level) and exposure to factors potentially related to STIs and/or BBDs (sexual behavior and past and current injecting drug use). The recall period for sexual behaviors and history of injecting drug use was lifetime.

### Clinical management of participants

All participants with confirmed HIV, HCV, HSV-2, and syphilis underwent a clinical evaluation. In cases presenting with HIV and HCV, patients were referred to the Geneva University Hospitals for specialized care. HCV-positive patients whose length of incarceration allowed doctors to consider possible treatment (interferon at the time of the study) benefited from a viremia and a liver biopsy. In accordance with the principle of equivalence of care, the indications for treatment were the same as those used in the general Swiss population. Participants with latent syphilis were treated with intramuscular penicillin (three times once a week). Participants with HSV-2 and recurrent genital ulcers received a prescription for antiviral medication to reduce symptoms during the next recurrent infection or antiviral prophylactic treatment (according to the frequency of relapses). All positive participants were also educated to limit the risk of transmission.

### Statistical analysis

Associations between infections (HCV, HSV-2) and other categorical variables (age, gender, region of origin, being an injecting drug user [IDU], and sexual health characteristics) were tested using Chi-square tests. We also computed Cramer’s V to provide an overview of the importance of the relationships for significant associations. The Cramer’s V ranges from 0 (no association) to 1 (perfect association), with .10 being a small association, .30 a medium association, and .50 a high association. Two multivariate logistic regressions that used HVC and HSV-2 as dependent variables and categorical factors as independent variables were performed. Stepwise regressions including variables (i.e., age, gender, region of origin, being an IDU, and sexual health characteristics) used in the bivariate analyses were performed. Odd ratios are reported. For HIV and syphilis, only descriptive statistics were computed because of the low prevalence rate of infected participants (*n* = 1 and *n* = 4 respectively). Descriptive and bivariate analyses were performed for the whole sample and then separately for 2009 and 2011 to test whether there were significant differences between the two time points. All analyses were performed using SPSS (version 24).

## Results

Table 1 summarizes the socio-demographic characteristics of the all-male participants. The mean age was 29.8 ± 9.0 years. The age range was 18–64 years, with 25% of the participants being younger than 23, 50% younger than 28, and 75% younger than 34. A total of 90.9% were migrants originating from outside Western Europe, and 63.7% were undocumented (no Swiss or European Union passport or residence permit in Switzerland). Overall, 72.1% attended secondary school (among all participants, 50.6% completed secondary school).

**Table 1:** Socio-demographic characteristics

Variable		N (%)	2009	2011	*p*-value^a^
Sex (male)		273 (100)	116 (100)	157 (100)	–
Region of origin^b^					
	Central and Eastern Europe	104 (38.1)	41 (35.3)	63 (40.1)	**.001**
	Sub-Saharan Africa	77 (28.2)	37 (31.9)	40 (25.5)	
	North Africa	39 (14.3)	26 (22.4)	13 (8.3)	
	Latin America	26 (9.5)	3 (2.6)	23 (14.6)	
	Western Europe	25 (9.2)	8 (6.9)	17 (10.8)	
	Asia	2 (0.7)	1 (0.9)	1 (0.6)	
Age					
	< 28 years	131 (48.0)	63 (54.3)	68 (43.3)	.072
	≥ 28 years	142 (52.0)	53 (45.7)	89 (56.7)	
Education level (8 missing values)					
	Secondary school not completed	74 (27.9)	78 (69.0)	113 (74.3)	.340
	Secondary school completed	191 (72.1)	35 (31.0)	39 (25.7)	
Self-reported socioeconomic status (8 missing values)					
	Low	43 (16.2)	101 (87.1)	121 (79.6)	**.033**
	Intermediate or high	222 (81.3)	12 (10.3)	31 (20.4)	

^a^Chi-square tests or Fisher exact tests. Significant *p*-values are highlighted in bold

^b^Countries of origin: Central and Eastern Europe: Albania (*n* = 44), Byelorussia (*n* = 3), Bosnia (*n* = 2), Georgia (*n* = 9), Israel (*n* = 1), Kosovo (*n* = 24), Lithuania (*n* = 1), Macedonia (*n* = 5), Romania (*n* = 6), Russia (*n* = 3), Serbia (*n* = 5), Slovakia (*n* = 1); Sub Saharan Africa: Angola (*n* = 4), Benin (*n* = 1), Cameroun (*n* = 2), Cap Verde (*n* = 1), Congo (*n* = 1), Eritrea (*n* = 2), Gambia (*n* = 1), Ghana (*n* = 3), Guinea (*n* = 7), Guinea Bissau (*n* = 2), Guinea Conakry (*n* = 20), Ivory Coast (*n* = 6), Liberia (*n* = 1), Mali (*n* = 3), Nigeria (*n* = 12), Senegal (*n* = 1), Sierra Leone (*n* = 5), Soudan (*n* = 1), Tanzania (*n* = 1), Togo (*n* = 2), Zimbabwe (*n* = 1); North Africa: Algeria (*n* = 15), Egypt (*n* = 1), Iraq (*n* = 2), Libya (*n* = 3), Morocco (*n* = 10), Palestine (*n* = 5), Tunisia (*n* = 3); Latin America: Brazil (*n* = 4), Bolivia (*n* = 1), Chili (*n* = 1), Colombia (*n* = 6), Dominican Republic (*n* = 12), Equateur (*n* = 1), Paraguay (*n* = 1); Western Europe: Belgium (*n* = 1), Germany (*n* = 1), Greece (*n* = 1), Italia (*n* = 2), France (*n* = 8), Portugal (*n* = 1), Spain (*n* = 1), Switzerland (*n* = 9), UK (*n* = 1); Asia: China (*n* = 1), India (*n* = 1)

In terms of sexual health, 2.3% of participants reported homosexual or bisexual behavior. More than half (52.8%) reported having had sexual activities with sex workers. Two-thirds (67.0%) reported occasional or no condom use. Almost three-quarters (73.0%) reported having had more than five sexual partners in their lifetime, and 5.9% of the participants declared a history of injecting drug use.

There were some differences between the two years of data collection: participants were more likely to come from North Africa in 2009 and Latin America in 2011, and the participants’ socio-economic level was lower in 2011. There were no differences for other risk factors included in the analyses presented below (history of sexual intercourse with sex workers, being an IDU, and history of genital ulcers).

Table 2 shows the prevalence of infections among participants. Antibody prevalence of HIV, HCV, syphilis, and HSV-2 were 0.4%, 6.2%, 1.1%, and 22.4%, respectively. There were no significant differences between 2009 and 2011. Only HSV-2 was comorbid with other infectious diseases: one participant also had HIV, two had syphilis, and one had HCV. There was no case of co-infection between HIV, syphilis, and HCV.

**Table 2:** Serological prevalence of HIV, HCV, syphilis and HSV-2

	n	% (95% CI)	2009	2011	*p*-value^a^
HIV (Ag/Ab Combo+ & Inno-Lia+)	1/270	**0.4%** (0.1–2.1)	0/115 (0%)	1/155 (0.7%)	1
Syphilis (ELISA+ & TPHA+ & RPR+)	3/271	**1.1%** (0.4–3.2)	2/114 (1.8%)	2/157 (1.3%)	1
HSV-2 (ELISA HSV-2+)	35/156	**22.4%** (16.6–29.6)	–	35/156 (22.4%)	–
HCV (EIA+ & Inno-Lia+)	17/273	**6.2%** (3.9–9.7)	8/116 (6.9%)	9/157 (5.7%)	.694

95% CI: 95% confidence interval

^a^Chi-square tests or Fisher exact tests. Significant *p*-values are highlighted in bold

A total of 76.5% of participants with HCV were aware of their infection before screening. Among HCV-positive participants who were aware of their status before screening, 92.5% reported being IDUs, whereas 25% of participants who were HCV-positive and unaware of their status before screening reported being IDUs, representing a statistically significant difference (*p* = 0.04). The HIV positive participant was aware of his infection before screening.

According to bivariate analysis, associations of HCV positivity with a history of being an IDU, originating from European countries, having sexual intercourse with sex workers, and having a low education level were statistically significant. We found an important association between HCV and injecting drug use. The other associations were small (max. = .181) (Table 3). Results showed no association between HCV-positivity and age, self-reported socioeconomic level, non-use of condoms, same-sex sexual activities, and the number of sexual partners in a participant’s lifetime. In the multivariate model, only IDUs were significantly associated with HCV. Participants who were IDUs were 227 times more likely to have HCV compared with participants who were not IDUs (*p* < .001).

**Table 3:** Bivariate and multivariate analyses of HCV and HSV-2 according to sociodemographic and sexual health factors

		HCV			HSV-2		
Variable ^c^		*n* = 17	*p*-value bivariate model	*p*-value multivariate model^d^	*n* = 35	*p*-value bivariate model	*p*-value multivariate model^d^
			V-Cramer	OR		V-Cramer	OR^d^
Region of origin							
	European countries	14/129 (**10.9%**)	**.003** ^a^		12/79 (**15.2%**)	**.028** ^a^	
	Other regions	3/144 (**2.0%**)	0.181		23/77 (**29.8%**)	0.176	
Age							
	< 28 years	5/131 (3.8%)	.113^a^		10/67 (14.9%)	.051^a^	**.019**
	≥ 28 years	12/142 (8.5%)	–		25/89 (28.1%)	0.156	2.63
Education level							
	Secondary school not completed	1/74 (**1.4%**)	**.047** ^b^		15/39 (**38.5%**)	**.009** ^a^	**.034**
	Secondary school completed	16/191 (**8.4%**)	0.129		20,112 (**17.9%**)	0.214	3.21
Condom use for sexual protection							
	Always	3/87 (3.5%)	.165^a^		16/63 (25.4%)	.585^a^	
	Sometimes or never	14/177 (7.9%)	–		19/88 (21.6%)	–	
Sexual intercourse with sex workers							
	Never	4/125 (**3.2%**)	**.044** ^a^		13/65 (20%)	.421^a^	
	At least once	13/140 (**9.3%**)	0.124		22/86 (25.6%)	–	
Sexual orientation							
	Homosexual/bisexual	1/6 (16.7%)	.662^b^		0/3 (0%)	.999^b^	
	Heterosexual	16/259 (6.2%)	–		35/148 (23.6%)	–	
Number of sexual partners							
	0 to 5	3/71 (4.2%)	.369^a^		8/44 (18.2%)	.450^a^	
	6 or more	14/192 (7.3%)	–		25/105 (23.8%)	–	
Injection drug use							
	Yes	13/16 (**81.2%**)	**<.001** ^b^	**<.001**	0/7 (0%)	.138^a^	
	No	4/253 (**1.6%**)	0.774	227.00	35/145 (24.1%)	–	
History of genital ulcers							
	Yes	2/28 (7.1%)	.695^b^		7/15 (**46.7%**)	**.045** ^b^	
	No	15/239 (6.3%)	–		28/138 (**20.3%**)	0.187	

95% CI: 95% confidence interval, OR: odd-ratio

^a^Chi square tests and ^b^ Fisher exact tests were performed with Cramers’ V as effect size

^c^No statistically significant association between HCV or HSV-2 and socio-economic status, condom use, same-sex sexual activities, and number of sexual partners lifetime (*p* > .05). No inferential statistics were performed on HIV and syphilis because of the low prevalence rate of infected people living in detention

Significant p-values and associated prevalence rates are highlighted in bold

^d^Multiple logistic stepwise regressions with HCV and HSV-2 as dependent variables were performed. *p*-values of Wald chi-square and odd-ratio with the second category of each variable being the reference category are reported

Bivariate analyses showed that HSV-2 was significantly associated with education level, region of origin, history of genital ulcers, and marginally associated with age. As for region of origin, 29.8% of participants from Africa and Latin America were seropositive against 15.2% of those originating from European countries. All effect sizes were small (max. = .214), with the highest being between HSV-2 and education level (Table 3). Results showed no association between HSV-2 infection and self-reported socioeconomic level, non-use of condoms, same-sex sexual activities, number of sexual partners in a participant’s lifetime, and sexual intercourse with sex workers. In the multivariate model, level of education and age were significantly associated with HSV-2. Participants with a low educational level (respectively, younger) were 3.21 (respectively 2.63) times more likely to have HSV-2 compared to participants with a higher level of education (respectively, older).

## Discussion

This study aimed to estimate the prevalence rate of HIV, syphilis, HCV, and HSV-2 in a sample of male detainees and to investigate associated risk factors. Overall, we found a high prevalence of HIV, HCV, syphilis, and HSV-2. Findings on hepatitis B are reported in a previous study (prevalence rate = 5.9%) [21].

### Prevalence rates of infectious diseases

The prevalence of HIV (0.4%) was two times higher in comparison with the general Swiss population (0.2%) [22]. However, this prevalence of 0.4% may be seen as low when considering the profile of the detainees (large proportion of migrants and multiple drug use) and in comparison with the prevalence found in other European prisons. For example, a previous study [23] established that the HIV prevalence in detainees in developed countries ranges from 0.2% in Australia to more than 10% in some European nations. The countries with the highest prevalence rates were in sub-Saharan Africa and Eastern Europe [12].

The prevalence of HCV (6.2%) was nine times higher in comparison withthe general Swiss population (0.71%) [24], but 2.5-fold lower than the average (15.5%) reported in prisons in Western European countries [12].

HSV-2 prevalence (22.4%) was more than two times higher in comparison with the Swiss general population of men of similar age (10.6% among 25–34-year-old participants) [25]. Studies investigating HSV-2 seroprevalence in prison are scarce. Among male detainees in Italy and Portugal, two studies reported an HSV-2 seroprevalence (21.0% and 19.9%) close to that reported in our study population [26]. In one prison in New South Wales, Australia, the prevalence of HSV-2 was estimated to be 21% among males [27].

Finally, the prevalence of syphilis among participants (1.1%) was sevenfold higher in comparison with the general population in European countries (0.16%) [28]. This high prevalence rate suggested that future studies should explore the cost-effectiveness of systematic screening. Indeed, it is estimated that a third of people infected with latent syphilis will present with significant complications if left untreated [29]. Syphilis remains a frequent infectious disease in low- and middle-income countries, and its prevalence rate has recently increased in Western countries [30]. Therefore, screening and treatment of such high-risk populations as detainees would help to reduce this burden of disease.

### Risk factors for HCV and HSV-2

We investigated risk factors for the two infectious diseases with a sufficient sample size for analysis. The factor most strongly associated with HCV was the history of drug injection (Cramer’s V = .774). This was also the only factor associated with HCV in the multivariate analysis. This observation was consistent with earlier findings [31], which showed that the HCV prevalence among detainees was higher compared to the general population due to the high proportion of IDU. The prevalence of HCV was lower among detainees originating from Africa, Latin America, and Asia than among those from Europe, a higher proportion of whom are IDUs. The proportion of migrants from Africa and Latin America, who are rarely IDUs (only 1.2%), is higher among detainees in Switzerland than in other Western European countries; this fact may explain the lower prevalence of HCV in Swiss prisons than in other Western European countries.

HCV-positive IDUs were more aware of their status (92.5%) than HCV-positive participants who did not inject drugs (25%). Therefore, the subgroup of detainees who did not inject drugs should not be neglected. Complementary studies targeting this subgroup are necessary to investigate HCV epidemiology, particularly among participants from countries with high HCV prevalence and where transmission is not predominantly due to injection drug use. Prisons should be a place that offers screenings and treatment for HCV to this vulnerable population, as members of this population are often hard to reach outside the prison. A study has shown that HCV treatment of detainees is cost-effective [32].

A low education level and increased age were the main risk factors associated with HSV-2 (as well as in the multivariate analysis). These factors corresponded to those identified in the literature [33]. Region of origin and a history of genital ulcers were significant only in the bivariate analyses. Participants from Africa and Latin America with HSV-2 positivity were twice as prevalent as European participants. According to Looker et al., the global burden of HSV-2 varies by region. The HSV-2 prevalence among 15–49-year-old men is the highest in Africa (25%), followed by the Americas (10%), Southeast Asia (7%), and European countries (4%) [34]. However, the region of origin was no longer significant in the multivariate analysis. This was probably due to a multicollinearity with socio-economic level, because migrants originating from low-income countries were likely to have a low educational level.

Factors related to sexual risk history did not efficiently discriminate HSV-2-infected detainees, but history of genital ulcers was associated with HSV-2 in bivariate analyses. Nevertheless, 79% of HSV-2-positive participants did not report a history of genital ulcers. This proportion is consistent with data reported in the literature, where only 9–25% of people who are HSV-2 positive report a history of symptoms suggestive of genital herpes [35]. Among our HSV-2-positive participants, 15% reported recurrent genital ulcers. Four out of five participants with recurrent genital ulcers did not seek medical care because they thought that no treatment was available. These people could be treated with antiviral medication to help reduce symptoms during recurrent infections, while antiviral prophylactic treatment could be prescribed to limit contagiousness and the frequency of relapses [8]. Moreover, these patients need to be educated regarding their contagiousness during and outside symptomatic episodes [36].

HSV-2 was also the only sexual infection associated with other diseases. The participant infected with HIV also had HSV-2, as did the two participants who had syphilis. The number of cases of HIV and syphilis was too small to conclude regarding the comorbidity between sexual infections, and future studies should focus on this important research question.

### Strengths and limitations

The strengths of our study included the high participation rate and the use of reliable indicators necessary for planning preventive measures. However, this study had some limitations. First, it included a relatively low number of participants and a limited number of positive cases; thus, it is difficult to make a definite statement about the risk factors, particularly for HIV and syphilis. Nevertheless, the sample size was sufficient to identify the burden of BBDs and STIs in the study population, as well as factors associated with HCV and HSV-2. Second, this study focused on men, so data among women are needed, even if women represent only 5% of the total prison population in Switzerland. Third, because participants were asked to answer sensitive questions (e.g., sexual practices), this information may be less reliable due to reporting bias, social desirability [37], and because the questionnaire was completed face-to-face with a physician. Fourth, for HCV and HSV-2, only serological markers were tested, indicating previous contact with the virus, but not necessarily current infection. Fifth, we used a self-reported measure of socioeconomic status. Future studies should use more reliable ways to assess this important factor. Finally, our findings may not necessarily be generalizable to detention centers in other countries. We hypothesize, however, that the profiles of infections described here would be comparable to other detention centers, where the sociodemographic profiles correspond to those described here. The study population was similar to other pre-trial prisons in Switzerland, which are also characterized by high proportions of migrants and males [15].

### Recommendations

This study highlighted some gaps in the policies designed to fight infectious diseases in Swiss prisons. As the burden of HIV, HCV, and STIs was high in this Swiss prison and because risk factors contributed to a higher risk of BBD transmission among prisoners [38], effective measures should be improved to mitigate BBD and STI transmission. Effective and internationally recommended strategies, such as opioid substitution treatment, condom distribution, and needle and syringe exchange programs [39], must be continued in the population study and introduced in other Swiss prisons where they are not yet enforced. Safer tattooing strategies should also be implemented, as tattooing is currently done in a clandestine and unsafe way by using inappropriate handcrafted equipment. Moreover, inmates share these tattoo devices, enhancing the risk of BBD transmission [37, 40]. Identification of BBDs and STIs must be strengthened in detention facilities in a way that makes it possible to ethically screen as many people as manageable, ensuring individual autonomy and access to treatment [39, 41].

Overall, we recommend strengthening preventive strategies in correctional settings, especially for detainees with a low education level and from countries with high HSV-2 endemicity [34]. Region of origin, even if non-significant in the multivariate model, is an important factor when screening for disease at entry to a prison. The socio-economic level is not assessed, whereas the region of origin is. Therefore, this factor might be used as an indicator of the likelihood for the presence of HSV-2. For this subgroup, we also recommend integrating genital ulcers into the participants’ medical history and strengthening preventive measures, such as encouraging people to use condoms.

## Conclusions

The prevalence of BBDs and STIs found in our incarcerated population was worryingly high. Screening, educational, and preventive programs to promote low-risk and health-seeking behaviors, as well as access to quality treatment and care should be guaranteed to detainees [42]. Infection control should be an important health care focus in prison to prevent possible health complications and further transmission of infectious diseases. Reducing BBD- and STI-related morbidity and breaking the existing transmission chain in prison settings would eventually benefit the larger communities into which detainees will be reintegrated [43].

## References

[1] Hammett TM (2006). HIV/AIDS and other infectious diseases among correctional inmates: transmission, burden, and an appropriate response. Am J Public Health.

[2] Negro F (2014). Epidemiology of hepatitis C in Europe. Dig Liver Dis.

[3] Felisberto M, Saretto AA, Wopereis S, Treitinger A, Machado MJ, Spada C (2016). Prevalence of human immunodeficiency virus infection and associated risk factors among prison inmates in the City of Florianópolis. Rev Soc Bras Med Trop.

[4] Fakoya I, Álvarez-del Arco D, Woode-Owusu M, Monge S, Rivero-Montesdeoca Y, Delpech V, Rice B, Noori T, Pharris A, Amato-Gauci AJ (2015). A systematic review of post-migration acquisition of HIV among migrants from countries with generalised HIV epidemics living in Europe: mplications for effectively managing HIV prevention programmes and policy. BMC Public Health.

[5] Carmona-Gutierrez D, Kainz K, Madeo F (2016). Sexually transmitted infections: old foes on the rise. Microb Cell.

[6] Stamm LV (2016). **Syphilis: Re-emergence of an old foe**. Microb Cell.

[7] Looker KJ, Elmes JAR, Gottlieb SL, Schiffer JT, Vickerman P, Turner KME, Boily M-C (2017). Effect of HSV-2 infection on subsequent HIV acquisition: an updated systematic review and meta-analysis. Lancet Infect Dis.

[8] Schiffer JT, Corey L (2009). New concepts in understanding genital herpes. Curr Infect Dis Rep.

[9] CDC: STD Facts - Syphilis (Detailed). In*.* Atlanta, USA: Centers for Disease Control and Prevention; 2017.

[10] Marques NMS, Margalho R, Melo MJ, da Cunha JGS, Meliço-Silvestre AA (2011). Seroepidemiological survey of transmissible infectious diseases in a Portuguese prison establishment. Braz J Infect Dis.

[11] Scott JA, Chew KW (2017). Treatment optimization for HIV/HCV co-infected patients. Ther Adv Infect Dis.

[12] Dolan K, Wirtz AL, Moazen B, Ndeffo-Mbah M, Galvani A, Kinner SA, Courtney R, McKee M, Amon JJ, Maher L (2016). Global burden of HIV, viral hepatitis, and tuberculosis in prisoners and detainees. Lancet (London, England).

[13] UNAIDS: The gap report 2014. In*.* Geneva, Switzerland: The gap report 2014; 2014.

[14] Kouyoumdjian FG, Leto D, John S, Henein H, Bondy S (2012). A systematic review and meta-analysis of the prevalence of chlamydia, gonorrhoea and syphilis in incarcerated persons. Int J STD AIDS.

[15] Office SFS: Swiss statistics - prisons, detention - key figures. In*.*: Swiss federal statistical Office; 2011.

[16] Rieder JP, Bertrand D, Wolff H, Gravier B, Pasche C, Bodenmann P (2010). Santé en milieu pénitentiaire : vulnérabilité partagée entre détenus et professionnels de la santé. Rev Méd Suisse.

[17] Baggio Stéphanie, Gétaz Laurent, Tran Nguyen, Peigné Nicolas, Chacowry Pala Komal, Golay Diane, Heller Patrick, Bodenmann Patrick, Wolff Hans (2018). Association of Overcrowding and Turnover with Self-Harm in a Swiss Pre-Trial Prison. International Journal of Environmental Research and Public Health.

[18] Wolff H, Sebo P, Haller DM, Eytan A, Niveau G, Bertrand D, Gétaz L, Cerutti B (2011). Health problems among detainees in Switzerland: a study using the ICPC-2 classification. BMC Public Health.

[19] Barro J, Casillas A, Gétaz L, Rieder J-P, Baroudi M, François A, Broers B, Wolff H (2014). Retractable syringes in a Swiss prison needle and syringe exchange program: experiences of drug-using inmates and prison staff perceptions. Int J Ment Health Addiction.

[20] Favrod-Coune T, Baroudi M, Casillas A, Rieder J-P, Gétaz L, Barro J, Gaspoz J-M, Broers B, Wolff H (2013). Opioid substitution treatment in pretrial prison detention: a case study from Geneva, Switzerland. Swiss Med Wkly.

[21] Gétaz L, Casillas A, Siegrist C-A, Chappuis F, Togni G, Tran NT, Baggio S, Negro F, Gaspoz J-M, Wolff H (2018). **Hepatitis B prevalence, risk factors, infection awareness and disease knowledge among inmates: a cross-sectional study in Switzerland’s largest pre-trial prison**. J Glob Health.

[22] Kohler P, Schmidt AJ, Cavassini M, Furrer H, Calmy A, Battegay M, Bernasconi E, Ledergerber B, Vernazza P, Swiss HIVCS (2015). The HIV care cascade in Switzerland: reaching the UNAIDS/WHO targets for patients diagnosed with HIV. AIDS.

[23] Hellard ME, Aitken CK (2004). HIV in prison: what are the risks and what can be done?. Sex Health.

[24] Sakem B, Madaliński K, Nydegger U, Stępień M, Godzik P, Kołakowska A, Risch L, Risch M, Zakrzewska K, Rosińska M (2016). Hepatitis C virus epidemiology and prevention in polish and Swiss population - similar and contrasting experiences. Ann Agric Environ Med.

[25] Bünzli D, Wietlisbach V, Barazzoni F, Sahli R, Meylan PRA (2004). Seroepidemiology of herpes simplex virus type 1 and 2 in Western and southern Switzerland in adults aged 25-74 in 1992-93: a population-based study. BMC Infect Dis.

[26] Sarmati L, Babudieri S, Longo B, Starnini G, Carbonara S, Monarca R, Buonomini AR, Dori L, Rezza G, Andreoni M (2007). Human herpesvirus 8 and human herpesvirus 2 infections in prison population. J Med Virol.

[27] Butler T, Donovan B, Taylor J, Cunningham AL, Mindel A, Levy M, Kaldor J (2000). Herpes simplex virus type 2 in prisoners, New South Wales, Australia. Int J STD AIDS.

[28] Newman L, Rowley J, Hoorn SV, Wijesooriya NS, Unemo M, Low N, Stevens G, Gottlieb S, Kiarie J, Temmerman M (2015). Global estimates of the prevalence and incidence of four curable sexually transmitted infections in 2012 based on systematic review and global reporting. PLoS One.

[29] Ficarra G, Carlos R (2009). Syphilis: the renaissance of an old disease with oral implications. Head Neck Pathol.

[30] Barnett R (2018). Syphilis. Lancet.

[31] Vescio MF, Longo B, Babudieri S, Starnini G, Carbonara S, Rezza G, Monarca R (2008). Correlates of hepatitis C virus seropositivity in prison inmates: a meta-analysis. J Epidemiol Community Health.

[32] Tan JA, Joseph TA, Saab S (2008). Treating hepatitis C in the prison population is cost-saving. Hepatology.

[33] Wald A (2004). Herpes simplex virus type 2 transmission: risk factors and virus shedding. Herpes.

[34] Looker KJ, Magaret AS, May MT, Turner KME, Vickerman P, Gottlieb SL, Newman LM (2015). Global and regional estimates of prevalent and incident herpes simplex virus type 1 infections in 2012. PLoS One.

[35] Jolivet R, Sahli R, Meylan PRA (2001). Herpès génital : l’épidémie silencieuse ?. Rev Méd Suisse.

[36] Koelle DM, Wald A (2000). Herpes simplex virus: the importance of asymptomatic shedding. J Antimicrob Chemother.

[37] Moazen B, Saeedi Moghaddam S, Silbernagl MA, Lotfizadeh M, Bosworth RJ, Alammehrjerdi Z, Kinner SA, Wirtz AL, Bärnighausen TW, Stöver HJ (2018). Prevalence of drug injection, sexual activity, tattooing, and piercing among prison inmates. Epidemiol Rev.

[38] Ndeffo-Mbah ML, Vigliotti VS, Skrip LA, Dolan K, Galvani AP (2018). Dynamic models of infectious disease transmission in prisons and the general population. Epidemiol Rev.

[39] Rich JD, Beckwith CG, Macmadu A, Marshall BDL, Brinkley-Rubinstein L, Amon JJ, Milloy MJ, King MRF, Sanchez J, Atwoli L (2016). Clinical care of incarcerated people with HIV, viral hepatitis, or tuberculosis. Lancet (London, England).

[40] Tran NT, Dubost C, Baggio S, Gétaz L, Wolff H (2018). Safer tattooing interventions in prisons: a systematic review and call to action. BMC Public Health.

[41] Tavoschi L, Vroling H, Madeddu G, Babudieri S, Monarca R, Vonk Noordegraaf-Schouten M, Beer N, Gomes Dias J, O'Moore É, Hedrich D (2018). Active case finding for communicable diseases in prison settings: increasing testing coverage and uptake among the prison population in the European Union/European economic area. Epidemiol Rev.

[42] Nokhodian Z, Yazdani MR, Yaran M, Shoaei P, Mirian M, Ataei B, Babak A, Ataie M (2012). Prevalence and risk factors of HIV, syphilis, hepatitis B and C among female prisoners in Isfahan, Iran. Hepat Mon.

[43] Kazi AM, Shah SA, Jenkins CA, Shepherd BE, Vermund SH (2010). Risk factors and prevalence of tuberculosis, human immunodeficiency virus, syphilis, hepatitis B virus, and hepatitis C virus among prisoners in Pakistan. Int J Infect Dis.

